# Infection/inflammation-associated preterm delivery within 14 days of presentation with symptoms of preterm labour: A multivariate predictive model

**DOI:** 10.1371/journal.pone.0222455

**Published:** 2019-09-12

**Authors:** Emmanuel Amabebe, Steven Reynolds, Xiaoya He, Robyn Wood, Victoria Stern, Dilly O. C. Anumba

**Affiliations:** 1 Academic Unit of Reproductive and Developmental Medicine, University of Sheffield, Sheffield, England, United Kingdom; 2 Academic Unit of Radiology, University of Sheffield, Sheffield, England, United Kingdom; Azienda Ospedaliero Universitaria Ospedali Riuniti di Ancona Umberto I G M Lancisi G Salesi, ITALY

## Abstract

Multi-marker tests hold promise for identifying symptomatic women at risk of imminent preterm delivery (PTD, <37 week’s gestation). This study sought to determine the relationship of inflammatory mediators and metabolites in cervicovaginal fluid (CVF) with spontaneous PTD (sPTD) and delivery within 14 days of presentation with symptoms of preterm labour (PTL). CVF samples from 94 (preterm = 19, term = 75) singleton women with symptoms of PTL studied between 19^+0^–36^+6^ weeks’ gestation were analysed for cytokines/chemokines by multiplexed bead-based immunoassay, while metabolites were quantified by enzyme-based spectrophotometry in a subset of 61 women (preterm = 16, term = 45). Prevalence of targeted vaginal bacterial species was determined for 70 women (preterm = 14, term = 66) by PCR. Overall, 10 women delivered within 14 days of sampling. Predictive capacities of individual biomarkers and cytokine-metabolite combinations for sPTD and delivery within 14 days of sampling were analysed by logistic regression models and area under the receiver operating characteristic curve. *Fusobacterium* sp., *Mubiluncus mulieris* and *Mycoplasma hominis* were detected in more preterm-delivered than term women (*P*<0.0001), while, *M*. *curtisii* was found in more term-delivered than preterm women (*P*<0.0001). RANTES (0.91, 0.65–1.0), IL-6 (0.79, 0.67–0.88), and Acetate/Glutamate ratio (0.74, 0.61–0.85) were associated with delivery within 14 days of sampling (AUC, 95% CI). There were significant correlations between cytokines and metabolites, and several cytokine-metabolite combinations were associated with sPTD or delivery within 14 days of sampling (e.g. L/D-lactate ratio+Acetate/Glutamate ratio+IL-6: 0.84, 0.67–0.94). Symptomatic women destined to deliver preterm and within 14 days of sampling express significantly higher pro-inflammatory mediators at mid to late gestation. In this cohort, IL-6, Acetate/Glutamate ratio and RANTES were associated with delivery within 14 days of sampling, consistent with their roles in modulating infection-inflammation-associated preterm labour in women presenting with symptoms of preterm birth. Replication of these observations in larger cohorts of women could show potential clinical utility.

## Introduction

Preterm delivery (PTD, before 37 weeks of gestation) remains the dominant global cause of perinatal morbidity and mortality. Preterm labour (PTL) is suspected when a woman presents with frequent uterine contractions occurring at least once every 5–8 minutes, cervical dilation >2 cm and cervical effacement (≥50%) before 37 weeks’ gestation [1]. Approximately 45% of PTDs are preceded by spontaneous PTL, while ~30% follow preterm premature rupture of membranes (PPROM) [2]. Majority of women presenting with symptoms of PTL do not eventually deliver preterm and would benefit from better prognostication of those most likely to imminently deliver preterm [3]. Accurate identification of these pregnant women facilitates prompt clinical decision-making, maternal treatment with steroids to aid fetal lung maturation, minimises unnecessary hospitalisations, and improves triaging of patients to centres with optimal neonatal care facilities.

The most commonly employed clinical test for predicting imminent PTD are quantitative fetal fibronectin (qfFN) and insulin-like growth factor binding protein-1 (Actim Partus) [4–7], however, due to the heterogeneity of the pathophysiology of PTD, several studies have explored other maternal clinical, inflammation and biochemical markers to predict PTD within 7–14 days in women presenting with symptoms of PTL [3, 8]. Previously we showed that decreased cervicovaginal fluid (CVF) acetate, probably produced by vaginal mixed anaerobes in the absence of *Lactobacillus* dominance, appears a suitable “rule-out” marker for delivery within 2 weeks in symptomatic women [9, 10]. Low CVF glutamate is also associated with reduced vaginal fluid acidity and bacterial vaginosis (BV) [11], an established risk factor of PTD [12–14]. We have also recently demonstrated the predictive value of several CVF pro-inflammatory mediators, either singly or in combination with qfFN for spontaneous PTD (sPTD) in high risk women without symptoms of PTL [15]. Combining multiple potential markers implicated in the pathogenesis of sPTD may provide a more accurate clinical screening approach [7, 16, 17].

Therefore, to determine whether CVF metabolites and cytokines/chemokines assessment enhanced the prediction of sPTD and delivery within 14 days of presentation with symptoms suggestive of PTL, we investigated the relationship between these markers of infection/inflammatory and sPTD and delivery within 14 days of presentation. We hypothesised that symptomatic pregnant women who eventually deliver prematurely, within 14 days of presentation/sampling, will show higher CVF pro-inflammatory mediators and acetate/glutamate ratio compared to those that delivered afterwards. Furthermore, a multivariable approach may provide a more comprehensive analysis of the patho-mechanism of sPTD.

## Methods

In this study, CVF samples were obtained from 94 women presenting with symptoms of PTL (19^+0^–36^+6^ weeks’ gestation) at the Labour Ward Assessment Suite of the Jessop Wing Maternity Hospital, Sheffield, UK,—regular uterine contractions (at least once every 10 mins) and cervical dilatation <3 cm, intact fetal membranes and no clinical evidence of genitourinary tract infection, abnormal cervical cytology or vaginal bleeding. CVF samples were obtained prior to any vaginal examination or clinical intervention such as administration of antibiotics, tocolytics, steroids, or any vaginal pessary.

Study participants were enrolled between January 2014 and March 2017 and closely monitored until delivery outcomes were ascertained. Immediately after the swab samples were obtained, quantitative fetal fibronectin (qfFN), vaginal sonographic determination of cervical length (CL) as well as vaginal pH measurement were also performed as previously reported [9, 10, 18, 19].

These studies were approved by the Yorkshire and Humber (Sheffield) Committee of the UK National Research Ethics Service (REC Number 13/YH/0167).

### CVF sample collection and preparation

Through a sterile Cusco’s vaginal speculum, 2 high vaginal swabs were obtained from the posterior vaginal fornix of each pregnant woman with sterile Dacron swab (Deltalab Eurotubo 300263, Fisher Scientific, UK) at presentation. The swabs were processed as previously described [9, 10, 18, 19]. Briefly, immediately after collection, swabs were stored at -20°C and processed within 1–3 days. The swabs were processed by placing in a 1.5 μl microfuge tube containing 400 μl (bacterial DNA extraction) and 600 μl (metabolite analysis) isotonic Phosphate Buffered Saline (PBS). The microfuge tube containing the cut end of the swab in PBS was vortexed for 5 minutes to wash the CVF into solution. The swab tip was safely discarded, and the remaining solution was centrifuged for 3 minutes at 13,000 rpm after which the supernatant was aspirated into a fresh tube and preserved at -80°C until further analysis.

### CVF cytokine measurement

From the 400 μl CVF sample reserved for bacterial DNA extraction, 50 μl was aspirated and transferred into a new 1.5 μl microfuge tube and analysed for IL-1α, IL-1β, IL-2, IL-4, IL-6, IL-8, IL-10, IL-12p20, RANTES, TNF-r1, and IFN-γ by multiplexed bead-based immunoassay (BDTM Cytometric Bead Array, BD Biosciences, CA, USA). This was performed according to the BD CBA Human Soluble Protein Master Buffer Kit instruction (See text in S1 File). Briefly, standard solutions of each cytokine (for calibration curve), and 25 μl CVF samples were mixed with a 25 μl solution of capture beads-antibodies conjugate (left for 1 hour) and then 25 μl Phycoerythrin detection reagent (left for additional 2 hours) at room temperature. The samples were then washed, centrifuged individually, left for resuspension, and then transferred to the plate. Mean Fluorescence Intensity (MFI) were generated by a Life Technologies Attune Acoustic Focusing Cytometry and Attune NxT Cytometric Software v.2.1. Actual cytokine concentrations in each sample was extrapolated from the generated standard curve. Cytokine analyses were performed by staff blinded from patient’s clinical data and eventual delivery outcome.

### ^1^H-NMR Spectroscopy

From the CVF samples dissolved in 600 μl PBS, 400 μl containing 20 μl D_2_O was analysed by ^1^H-Nuclear Magnetic Resonance (NMR) spectroscopy using a 400 MHz (9.4T) Bruker Avance III NMR spectrometer (Bruker BioSpin GmbH, Karlsruhe, DE), with 5mm BBO probe. Lactate, acetate, glutamate and other metabolites implicated in vaginal host-microbial activities, infection and PTD were identified in the ^1^H-NMR spectrum (S1 Fig). Further analyses, integration and normalisation to obtain normalised integrals (n.i.) of identified metabolites were also performed as described previously [9, 10, 18, 19].

### CVF metabolite measurement by spectrophotometry

The NMR-guided metabolites were chosen as the basis for a cheaper clinically applicable test [20]. We measured absolute concentrations of metabolites by enzyme-based spectrophotometric assays (Megazyme, IE): acetate (K-ACETGK 08/14), glutamate (K-GLUT 07/12), L- and D-lactate (K-LATE 07/14 and K-DATE 04/14), glucose (K-GLUHK-110A/K-GLUHK-220A 07/14), pyruvate (K-PYRUV 04/14), urea (K-URAMR 07/17), and succinate (K-SUCC 01/14), from a randomly selected subgroup of 61 women (preterm = 16, term = 45, i.e. all women whose delivery outcomes where known at the time of analysis).). All measurements except pyruvate and urea were performed on the ChemWell^®^ 2910 auto-analyser (Awareness Technology, USA). Measurement of urea was performed on the ChemWell^®^-T auto-analyser (Awareness Technology, USA), while pyruvate measurement was on the MegaQuant wave spectrophotometer (Megazyme, IE).

### Polymerase chain reaction (PCR)

To identify the potential microbial composition/stimulus inducing the expression of metabolites and cytokines, the prevalence of targeted vaginal bacterial species was also determined for a random subgroup of 70 women (preterm = 14, term = 66) by PCR (i.e. all women whose delivery outcomes where known at the time of analysis). Bacterial 16S rDNA were extracted using the QIAamp DNA mini kit (Qiagen, UK) and amplified by genus/species-specific primers (Sigma-Aldrich, UK, S1 Table) [21–24], as previously described [15, 18]. Amplification was performed on an Applied Biosystems 2720 Thermal cycler (Life Technologies, UK) using a 25 μl reaction mix containing 12.5 μl AmpliTaq Gold DNA polymerase (Applied Biosystems, UK), 5 ng genomic DNA template, 1 μl each of 10 μM forward and reverse primers. The cycling criteria included 95°C (5 mins)–denaturation, followed by 35 cycles of 95°C (1 min)—denaturing, 50–62°C depending on the primer sets (1 min)–annealing, 72°C (1 min)–elongation, with a final extension at 72°C (7 mins). The amplicons were visualized/confirmed on a UV- transilluminator by 1% agarose gel electrophoresis stained with ethidium bromide. Positive results were assigned according to the presence of bands of appropriate size relative to the DNA marker. Bacterial 16S rDNA standards were also used as positive controls.

### Statistical analysis

Data were subjected to Shapiro-Wilk normality tests prior to analyses. Differences in maternal clinical data, CVF cytokine and metabolite concentrations between term- and preterm-delivered women as well as between women that delivered within and after 2 weeks of sampling were determined by Mann-Whitney *U* test. The relationships between cytokine, metabolite expression levels and maternal clinical and demographic variables were determined by nonparametric Spearman’s (rho) correlation coefficients. *P*-values < 0.05 were considered statistically significant. Bonferroni corrections were applied for multiple measurements and correlations. The differences in the prevalence of vaginal bacterial species between preterm- and term-delivered women were determined by Fisher’s exact test. Predictive capacities of CVF cytokine-metabolite combinations for sPTD (< 37 weeks of gestation) and delivery within 2 weeks of sampling were analysed by logistic regression models and area under the receiver operating characteristic curve (AUC). All analyses were performed using SPSS 24 (SPSS Inc., IL, USA), GraphPad Prism 7.03 (GraphPad Software, Inc. USA), and MedCalc 18.9 (MedCalc Software bvba, Ostend, BE; http://www.medcalc.org; 2018) statistical software packages.

## Results

### Maternal demographic and clinical details

Ninety-four women (preterm = 19, term = 75) with singleton pregnancies presenting with symptoms of PTL were enrolled in the study. Nineteen (20.2%) women delivered preterm, of which 10 (10.6%) delivered within 2 weeks of sampling. All women included in this study experienced spontaneous onset of labour, while those that underwent elective caesarean section without prior labour were excluded. Details of maternal demographic and clinical data are presented in Table 1. The preterm-delivered women had significantly higher BMI (*P* = 0.02) and qfFN (*P* = 0.005) and shorter CL (*P* = 0.002) compared to the term-delivered women. Most of the demographic and clinical characteristics were similar in women who delivered within and after 2 weeks of sampling, except that the women who delivered within 2 weeks of sampling were older (*P* = 0.03) than those that delivered later.

**Table 1:** Maternal clinical and demographic details according to delivery outcomes.

Characteristic	Term (N = 75)	Preterm (N = 19)	*P*-value	>2 weeks (N = 84)	<2 weeks (N = 10)	*P*-value
Age, years	26.8±5.7 (n = 70)	29.9±8.1 (n = 17)	0.2	26.9±5.8 (n = 74)	32.7±8.4 (n = 9)	**0.03**
BMI, kg/m^2^	25.4±5.0 (n = 60)	28.6±5.7 (n = 14)	**0.02**	25.8±5.4 (n = 65)	26.2±3.6 (n = 7)	0.4
GAAP, weeks	30±3.8 (n = 75)	28.8±3.2 (n = 19)	0.2	29.8±3.9 (n = 80)	29.5±2.8 (n = 10)	0.6
GAAD, weeks	39.0±1.2 (n = 71)	31.6±3.1 (n = 19)	**<0.0001**	38.3±2.3 (n = 80)	30.2±2.9 (n = 10)	**<0.0001**
qfFN, ng/ml	19.2±31.6 (n = 45)	215.5±227.8 (n = 10)	**0.005**	44.0±105.0 (n = 47)	187.6±233.1 (n = 5)	0.1
CL, mm	30.9±10.1 (n = 42)	18.4±13.4 (n = 12)	**0.002**	28.7±10.8 (n = 45)	18.7±17.4 (n = 6)	0.1
Vaginal pH	4.2±0.6 (n = 41)	4.3±0.8 (n = 11)	0.9	4.2±0.6 (n = 43)	4.6±0.9 (n = 6)	0.3

Data are presented as Median ± standard deviation (SD). Nine of the preterm women delivered after 2 weeks of sampling. *BMI*, body mass index; *qfFN*, quantitative fetal fibronectin; *CL*, cervical length; *GAAP*, gestational age at presentation; *GAAD*, gestational age at delivery. n = reduced study population where participants’ consent and/or data are absent.

### Prevalence of vaginal anaerobic bacterial species

**Fig 1:**
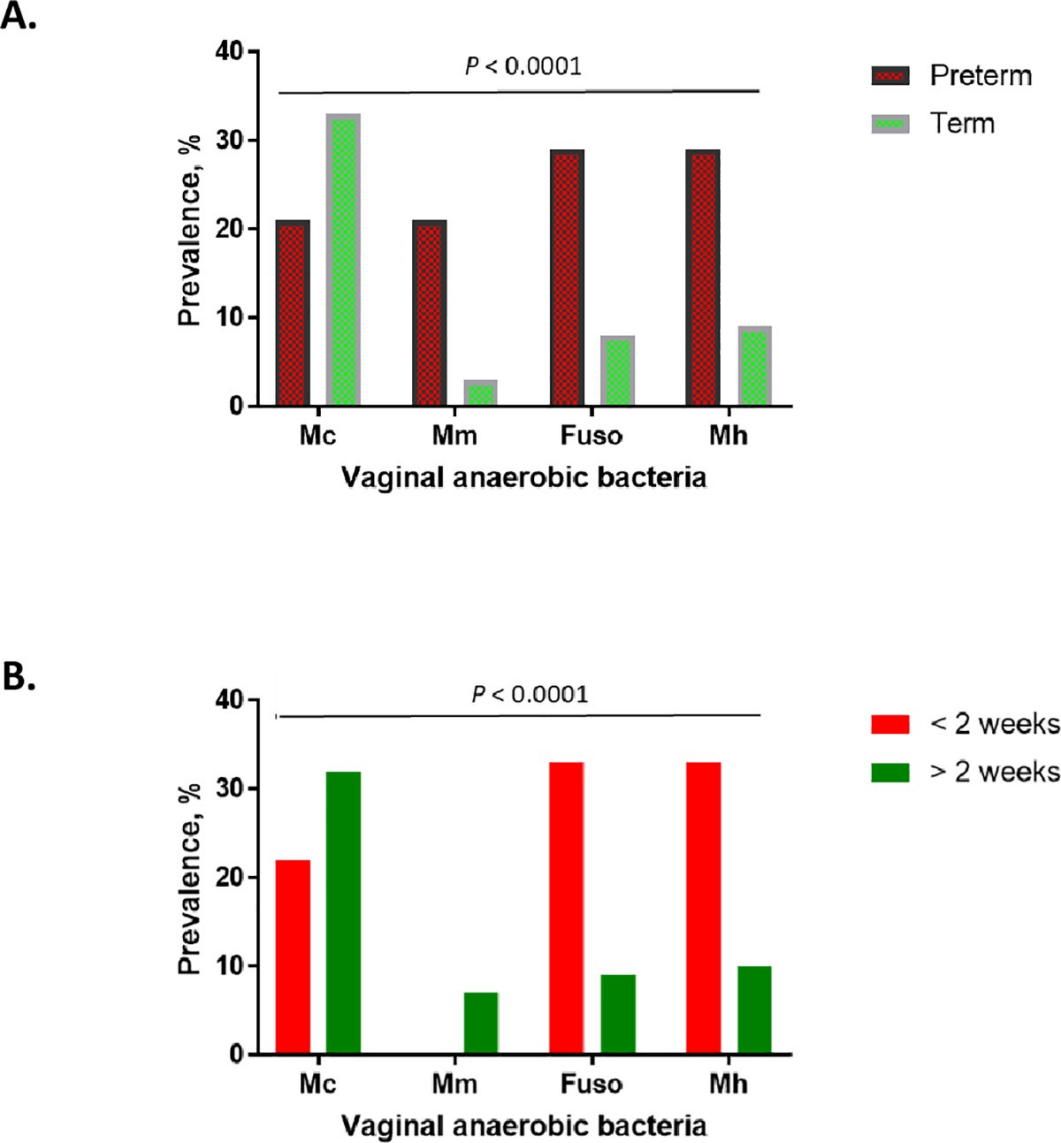
Prevalence of vaginal mixed anaerobic bacterial species in relation to delivery outcome. (A) Preterm vs. term. The preterm-delivered women had higher prevalence of *Fusobacterium* sp. (Fuso), *Mubiluncus mulieris* (Mm), *Mycoplasma hominis* (Mh), whereas *Mubiluncus curtisii* (Mc) was more prevalent in term-delivered women though to a lesser proportion. (B) Delivery within and after 2 weeks. *Fusobacterium* sp. and *M*. *hominis* were more prevalent in women who delivered within 2 weeks of sampling, whereas *M*. *curtisii* and *M*. *mulieris* were more prevalent in women that delivered later than 2 weeks of sampling.

The individual prevalence of *Fusobacterium* sp. (21%), *Mubiluncus mulieris* (18.4%) and *Mycoplasma hominis* (19.5%) was found to be higher in the preterm women compared to their term counterparts (*P* < 0.0001), while, *M*. *curtisii* was found in 11.6% more term-delivered than preterm women (*P* < 0.0001) (Fig 1). Furthermore, *Fusobacterium* sp. and *M*. *hominis* were more prevalent in women who delivered within 2 weeks of sampling, whereas *M*. *curtisii* and *M*. *mulieris* were more prevalent in women that delivered later than 2 weeks of sampling (*P* < 0.0001) (Fig 1). The prevalence of *Gardnerella vaginalis*, *Bacteroides* sp., and *Lactobacillus* sp., which were present in at least 77%, 48% and 98% of the women respectively regardless of the sub-grouping, did not differ significantly between the groups. Group B *Streptococcus* was only identified in one woman who delivered at term.

### CVF cytokine concentrations

CVF cytokine concentrations in relation to delivery outcomes are presented in Table 2. TNF-r1 (*P* = 0.006), RANTES (*P* = 0.02), IL-6 (*P* = 0.03) were significantly higher in preterm-delivered women compared to the term women. Additionally, RANTES (*P* = 0.02) and IL-6 (*P* = 0.03) were significantly higher in women who delivered within 2 weeks of sampling compared to those who delivered afterward. None of the other cytokines i.e. IL-1α and β, and IL-8 showed significant differences in relation to delivery outcomes between the cohorts, while IL-2, IL-4, IL-10, IL-12p70 and IFN-γ were below the detectable limit of the assay kit in all the samples and were omitted in subsequent analyses.

**Table 2:** Comparison of cervicovaginal fluid cytokine/chemokine concentrations (pg/ml) in relation to delivery outcomes.

**Cytokine/chemokine**	**Term (N = 75)**	**Preterm (N = 19)**	***P*-value**
**TNF-r1**	781.4 (283.3–1814.5) (n = 75)	2350.7 (645.7–3795.8) (n = 19)	**0.006**
**IL-1β**	369.5 (69.4–760.8) (n = 61)	785.6 (133.5–1653.4) (n = 9)	0.2
**RANTES**	40.8 (12.8–67.3) (n = 9)	101.4 (70.7–541.3) (n = 6)	**0.02**
**IL-6**	38.3 (6.1–147.2) (n = 47)	207.6 (47.1–524.5) (n = 13)	**0.01**
**IL-8**	771.6 (299.3–1385.3) (n = 20)	490.3 (402.5–1827.8) (n = 4)	0.8
**IL-1α**	546.2 (218.7–935.5) (n = 62)	1062.4 (397.6–1816.5) (n = 17)	0.3
**Delivery within 2 weeks of sampling**			
	**> 2 weeks (N = 84)**	**< 2 weeks (N = 10)**	***P*-value**
**TNF-r1**	922.5 (323.2–1936.5) (n = 80)	2083.8 (454.6–3187.0) (n = 10)	0.2
**IL-1β**	362.1 (75.2–1018.3) (n = 62)	628.8 (125.9–1883.5) (n = 4)	0.6
**RANTES**	40.8 (17.9–79.2) (n = 11)	289.1 (89.7–660.2) (n = 4)	**0.02**
**IL-6**	47.8 (6.1–147.2) (n = 51)	385.8 (56.8–592.9) (n = 7)	**0.03**
**IL-8**	490.3 (273.3–0.0) (n = 22)	490.3 (473.3–0.0) (n = 2)	0.4
**IL-1α**	583.6 (231.8–1138.7) (n = 67)	1062.4 (298.5–1940.9) (n = 9)	0.3

Data presented as median (25th - 75th percentile). Samples without a particular cytokine or cytokine concentration below the detectable limit of the assay kit (indicated as a value of zero) were omitted in subsequent analyses, hence the varied *n* numbers. Significant *P*-values are marked in bold.

### Metabolite concentration measured by spectrophotometry

**Fig 2:**
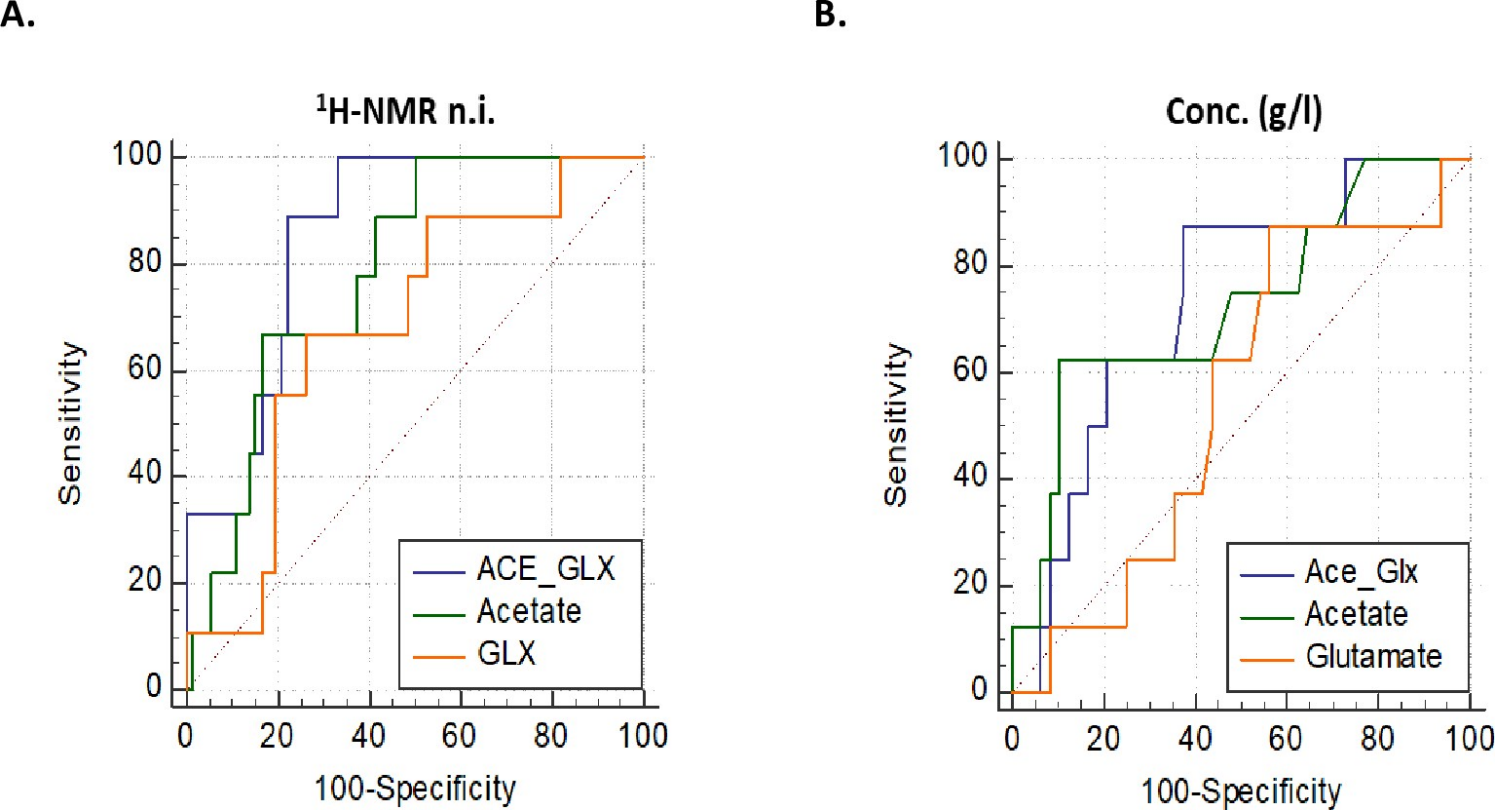
Receiver operating characteristic curve analysis of cervicovaginal fluid metabolites in predicting delivery within 2 weeks of presentation with symptoms of preterm labour. (A) ^1^H-NMR-derived acetate/glutamate ratio (Ace/Glx, AUC = 0.86, 0.76–0.93), Acetate (AUC = 0.78, 0.68–0.87) and Glutamate (AUC = 0.68, 0.57–0.78); (B) Metabolite concentrations measured by enzyme-based spectrophotometry: Ace/Glx (AUC = 0.74, 0.61–0.85), Acetate (AUC = 0.73, 0.59–0.84) and Glutamate (AUC = 0.54, 0.41–0.68). *n*.*i*., normalised integral.

We have previously shown good correlation between ^1^H-NMR and biochemical assay [10]. Table 3 shows differences in metabolite concentrations measured by spectrophotometry between study cohorts. Similar to ^1^H-NMR-derived normalised integrals, the absolute concentration of acetate was higher in the women who delivered within 2 weeks of sampling compared to those that delivered later (*P* = 0.047), as reported previously in a smaller population [10]. Although the glutamate concentration was not significantly different, the ratio (Ace/Glx_conc._) was more than 4-fold higher in the women who delivered within 2 weeks (*P* = 0.03) and was associated with delivery within 2 weeks of presentation with PTL (AUC = 0.74) (Table 3 and Fig 2). Predictive utility of spectrophotometry by ROC analysis was similar to the ^1^H-NMR-derived ratio (Ace/Glx_n.i_).

**Table 3:** Cervicovaginal fluid metabolites concentrations in relation to delivery outcome.

**Metabolites**	**Term**	**Preterm**	**AUC (95% CI)**	***P*-value**
**Acetate**	0.02 (0.01–0.03) n = 41	0.04 (0.02–0.08) n = 15	0.79(0.66–0.89)	**0.0007**
**Glutamate**	0.07 (0.04–0.10) n = 42	0.07(0.04–0.13) n = 15	0.53(0.40–0.67)	0.7
**Acetate/Glutamate ratio**	0.27 (0.14–0.50) n = 42	0.83 (0.30–1.89) n = 15	0.74 (0.60–0.85)	**0.006**
**Delivery within 2 weeks**				
	**> 2 weeks**	**< 2 weeks**	**AUC (95% CI)**	***P*-value**
**Acetate**	0.021 (0.01–0.03) (n = 47)	0.054 (0.02–0.08) (n = 8)	0.73 (0.59–0.84)	**0.047**
**Glutamate**	0.068 (0.04–0.10) (n = 49)	0.063 (0.05–0.08) (n = 8)	0.54 (0.41–0.68)	0.7
**Acetate/Glutamate ratio**	0.282 (0.16–0.58) n = 47	1.153 (0.40–1.77) n = 8	0.74 (0.61–0.85)	**0.03**

Concentrations of metabolites (g/l) derived by spectrophotometry are presented as median (25th and 75th percentiles). *AUC*, area under the ROC curve; *CI*, confidence interval.

There were no significant differences in the concentration of the L- and D- lactate, glucose, succinate, pyruvate, and urea between any of the cohorts, and none of these metabolites was predictive of PTD nor delivery within 2 weeks of sampling individually.

### Association of CVF cytokines, metabolites and maternal clinical data

**Fig 3:**
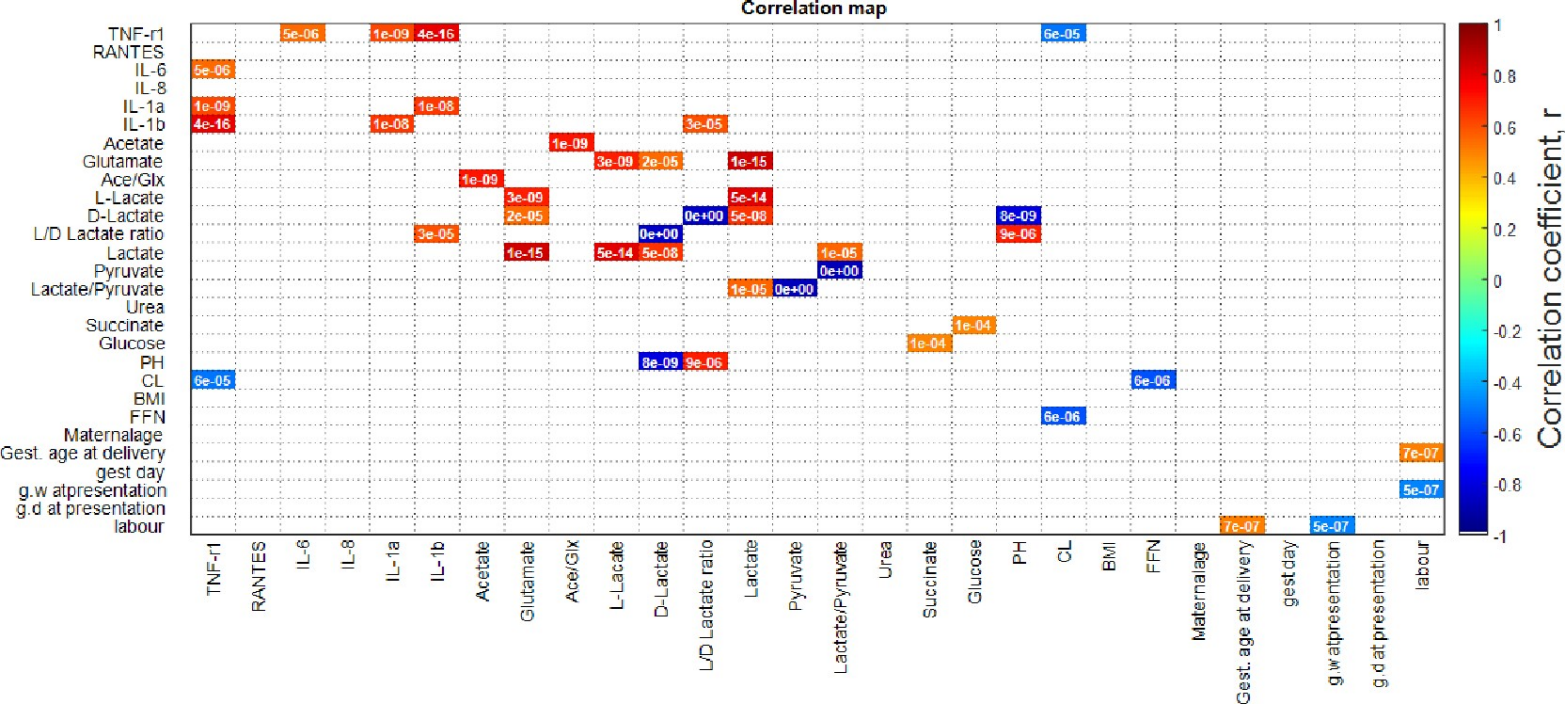
Correlation of cervicovaginal fluid pro-inflammatory mediators, metabolite concentrations and maternal clinical and demographic details. Spearman’s correlation coefficients (*r*, colour) range from -1 (dark blue) to +1 (dark red); and significant *P*-values (< 0.05) stated in the coloured boxes. Due to the high number (28) of compared measures, a Bonferroni corrected value of 0.05/364 = p<0.000137 was applied to minimise Type 1 error. *Ace/Glx*, acetate/glutamate ratio; *CL*, cervical length; *BMI*, body mass Index; *FFN*, quantitative fetal fibronectin; *Gest*., gestational; *g*.*w*, gestational week; *g*.*d*, gestational day; *labour*, latency i.e. interval between presentation and delivery.

The correlations between CVF pro-inflammatory mediators, metabolites (measured by enzyme-based spectrophotometry) and maternal clinical and demographic details are presented in Fig 3. After Bonferroni correction to minimise Type 1 error, TNF-r1 positively correlated with IL-1α, IL-1β, IL-6 and negatively with CL. IL-1β also correlated positively with L/D-lactate ratio which correlated positively with vaginal pH. Furthermore, D-lactate correlated positively with glutamate and inversely with vaginal pH. CL and qfFN were also negatively correlated.

### Receiver operating characteristic analysis of CVF biomarkers and clinical details for spontaneous PTB

As shown in S2 Table, RANTES, TNF-r1, IL-6, Ace/Glx_conc_, BMI, qfFN, which were significantly increased and CL, which was significantly decreased in the preterm women were able to determine risk of sPTD individually, but only RANTES, IL-6, Ace/Glx_conc_ and maternal age were associated with delivery within 2 weeks of sampling.

Furthermore, certain cytokine-metabolite combinations containing lactate, acetate, Ace/Glx_conc_, IL-6, and TNF-r1 were associated with sPTD, while others were associated with delivery within 2 weeks of sampling (S2 Table).

**Fig 4:**
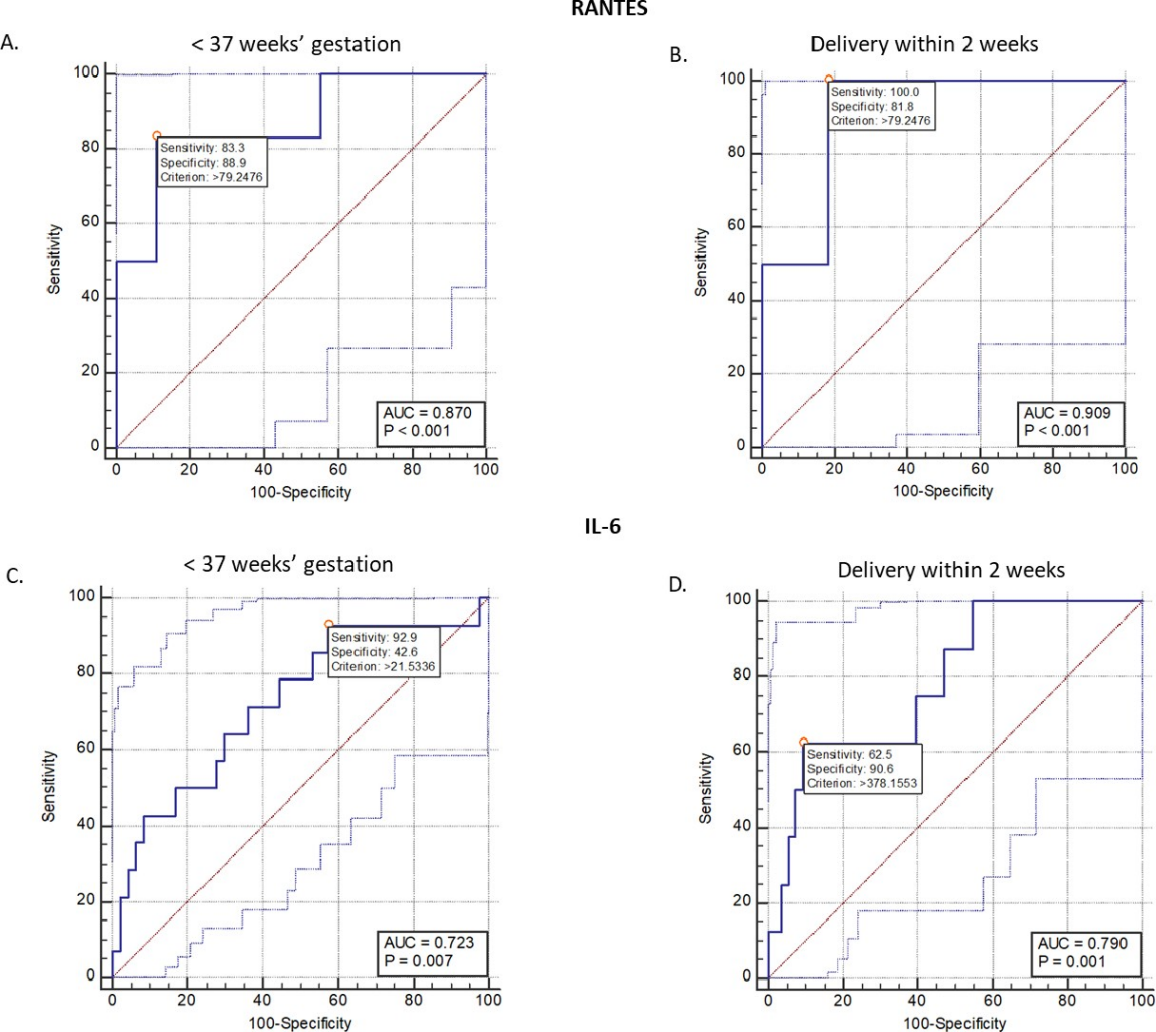
**Receiver operating characteristic curve analysis of cervicovaginal fluid RANTES (A-B) and IL-6 (C-D) in predicting preterm delivery and delivery within 2 weeks of presentation with symptoms of preterm labour.** *AUC*, area under the ROC curve.

RANTES, which had the highest individual predictive values for sPTD (AUC = 0.87) and delivery within 2 weeks of sampling (AUC = 0.91) (Fig 4 and S2 Table) could not be included in the combination (multibiomarker) models by logistic regression due to reduced number of samples with detectable RANTES concentrations.

## Discussion

This study investigated the relationship of CVF inflammatory mediators, microbiota metabolites and risk of imminent sPTD in women presenting with PTL. We observed that women who delivered preterm or within 2 weeks of sampling demonstrated higher prevalence of potentially pathogenic vaginal anaerobes in the CVF, and showed significantly higher markers of vaginal dysbiosis (Ace/Glx ratio), inflammation (RANTES, TNFr-1 and IL-6), shorter CL and increased qfFN. Cytokine-metabolite combinations of total lactate, L/D-lactate ratio, acetate, acetate/glutamate ratio, IL-6, TNF-r1 were also associated with sPTD and delivery within 2 weeks of presentation.

The observed cytokine-metabolite alterations were accompanied by high prevalence of anaerobes such as *Fusobacterium* sp., *M*. *hominis* and *M*. *mulieris* in women destined to deliver preterm; while those that delivered within 2 weeks of sampling showed higher prevalence of *Fusobacterium* sp. and *M*. *hominis* only. This is consistent with reports of elevated CVF IL-6 and positive cultures of *Ureaplasma urealyticum* and *M*. *hominis* in the chorioamniotic membranes in women with symptoms of PTL [25]. Our observations are also consistent with reports that vaginal communities dominated by anaerobes are potentially associated with greater pro-inflammatory cytokine responses than *Lactobacilli*-dominated niches [26, 27].

Increased vaginal concentrations of acetate and glutamate have contrasting implications for reproductive health. Acetate in the vaginal milieu is produced majorly by anaerobes, especially when *Lactobacilli* are deficient [28–30], and high amounts are associated with infection and PTD [9, 10, 18, 30]. Contrastingly, high vaginal glutamate level, similar to D-lactate, is associated with healthy microbiota dominated by *Lactobacilli* [11, 31] and decreased prevalence of anaerobes that are known to metabolise amino acids to their catabolic by-products [11] including acetate [18, 30]. For instance, *Fusobacterium* sp. which was more prevalent in the women who delivered preterm and within 2 weeks of sampling, ferments glutamate to acetate via the hydroxyglutarate pathway [32, 33]. Such metabolic processes explain our observation that the Ace/Glx ratio was associated with sPTD and delivery within 2 weeks of sampling. It is noteworthy that glutamate alone was not associated with delivery within 2 weeks, while acetate alone was to a lesser extent associated with imminent sPTD [9, 10]. Therefore, the Ace/Glx ratio was more strongly associated with delivery within 14 days of sampling than either acetate or glutamate individually. Furthermore, decreased glutamate, D-lactate, and increased L/D-lactate ratio were associated with high vaginal pH.

Despite our limited sample size, an association of CVF RANTES, IL-6 and TNF-r1 with sPTD within 14 days was also demonstrated. Additionally, the combination of metabolite indices (acetate, glutamate, L- and D-lactate) and cytokine concentrations were similarly associated with imminent sPTD, observations which are reflective of vaginal host-microbial interactions [34], being downstream products of gene expression and protein synthesis [35–37]. Our study gives additional insight into the role played by the vaginal ecosystem and host immune responses in the propagation of sPTD [38], and whether specific cytokine-metabolite combinations can be used to screen for women at high risk of imminent sPTD.

Elevated blood, serum and CVF RANTES at mid to late gestation has been associated with sPTD in both symptomatic [3, 37] and asymptomatic women [15, 39], as well as murine models [40]. We recently reported decreased CVF RANTES from mid to late second trimester in asymptomatic women who delivered at term compared to their preterm counterparts in whom levels remained unchanged [15]. RANTES is a potent chemoattractant and inducer of inflammatory mediators involved in parturition and is produced by immune, endothelial and gestational tissues [37, 41, 42].

Apart from RANTES, TNF-r1 (that binds TNF-α) and IL-6 were also increased in women who delivered preterm. IL-6 was additionally associated with delivery within 2 weeks. These pro-inflammatory mediators are implicated in the NF-κB-p38MAPK-orchestrated inflammatory cascade implicated in sPTL leading to myometrial contraction, cervical remodelling, and degradation/rupture of fetal membranes [31, 43–45]. TNF-α is detectable early in the amniotic fluid following bacterial colonisation and stimulates the production of IL-6, IL-8, matrix metalloproteinases and PGE_2_ during infection-associated PTL [42]. IL-6 activates the acute phase response [46] and, along with TNF-α, which were correlated in our study, correlates with qfFN [47] and is associated with PTD [48–50].

An altered vaginal microbiota with reduced *Lactobacillus* sp. dominance may increase vaginal pH creating a permissive environment for the proliferation of pathogenic anaerobes [51]. There is also a direct link between vaginal dysbiosis, short cervix [45, 52], and disrupted fetal membranes with consequent leakage of fFN into the cervicovaginal space [7, 45, 53]. Therefore, a high CVF Ace/Glx ratio could be suggestive of a dysbiosis with associated reduced D-lactate, glutamate and vaginal acidity, increased expression of pro-inflammatory mediators, shortened cervix and elevated qfFN as shown in this study and other reports [3, 15, 25, 30, 31, 45, 50].

Hitherto, no multivariable model of inflammatory mediators and cervical length associated with imminent sPTD in symptomatic [3, 54] and asymptomatic women [15, 55] has achieved clinical utility. Interestingly, we observed amongst others that combination of CVF: 1) acetate, lactate, IL-6 and TNF-r1; and 2) Ace/Glx ratio, L/D-lactate ratio and IL-6 were associated with sPTD and delivery within 14 days of sampling, respectively.

Two recent extensive systematic reviews of the accuracy of qfFN for the prediction of PTD within 7 to 10 days of testing in symptomatic women reported pooled sensitivities of 75% and 77%; and pooled specificities of 79% and 83% respectively [56, 57]. Both our single and multivariable cytokine-metabolite models on a similar cohort showed greater sensitivities and specificities for determining sPTD within 2 weeks of sampling respectively. However, there is the need to test these findings in larger and more diverse study populations.

## Conclusion

Symptomatic women destined to deliver preterm within 2 weeks express significantly higher pro-inflammatory mediators at mid to late gestation. In this cohort, RANTES, IL-6, and acetate/glutamate ratio; were more associated with imminent preterm delivery compared to other biomarkers. The combination of CVF metabolites and pro-inflammatory mediators appears able to distinguish symptomatic women at risk of imminent preterm birth. This approach may improve the triaging of such women, allowing patients to be discharged to outpatient care whilst sign-posting neonatal high-dependency care to those at highest risk. A larger study is required to confirm these observations and determine the cost utility of this approach in clinical care.

## Supporting information

S1 Fig Cervicovaginal fluid metabolite peaks identified on ^1^H-NMR spectrum at 400 MHz and 294K. *BCAA*, branched chain amino acids; *ppm*, parts per million.
(TIF)

S1 Table Primer sequences used for bacterial 16S rDNA amplification. *GBS*, group B *Streptococcus*.
(PDF)

S2 Table Predictive performance of biomarkers for imminent preterm delivery. *AUC*, area under the ROC curve; *CI*, confidence interval; *Sens*, sensitivity; *Spec*, specificity; *PPV*, positive predictive value; *NPV*, negative predictive value; *+LR*, positive likelihood ratio; *-LR*, negative likelihood ratio; *BMI*, body mass index; *CL*, cervical length; *qfFN*, quantitative fetal fibronectin; *Ace/Glx*_*conc*_, ratio of acetate to glutamate concentrations. All metabolite concentrations were measured by enzyme-based spectrophotometry.
(PDF)

S1 File CVF cytokine measurement. (PDF)

S1 Dataset CVF cytokines, metabolites and PCR data. (ZIP)

## Abbreviations

Ace/Glx: Acetate/Glutamate ratio
*a*.*u*.: arbitrary unit
AUC: Area under the Receiver Operating Characteristics (ROC) curve
BMI: Body mass index
BV: Bacterial vaginosis
CCL5: chemokine (C-C motif) ligand 5
CI: Confidence interval
conc: absolute concentration
CVF: Cervicovaginal fluid
EBS: Enzyme-based spectrophotometry
IFN-γ: interferon gamma
*IL*-: interleukin
NF-κB: nuclear factor-κB
*n*.*i*.: ^1^H-NMR-derived normalised integral
NMR: Nuclear Magnetic Resonance Spectroscopy
MAPK: mitogen-activated protein kinase
NPV: negative predictive value
q*fFN*: quantitative Fetal fibronectin
PGE_2_: prostaglandin E2
PPROM: Preterm premature rupture of membranes
PPV: positive predictive value
PTD: preterm delivery
PTL: preterm labour
RANTES: regulated on activation, normal T cell expressed and secreted
Sen: sensitivity
sIL-6Rα: soluble form of IL-6 receptor-α
Spec: specificity
sPTD: spontaneous preterm delivery
TNF-r1: tumor necrosis factor receptor 1
+LR: positive likelihood ratio
-LR: negative likelihood ratio

## References

[1] Bianchi-Jassir F, Seale AC, Kohli-Lynch M, Lawn JE, Baker CJ, Bartlett L, et al Preterm Birth Associated With Group B Streptococcus Maternal Colonization Worldwide: Systematic Review and Meta-analyses. Clinical Infectious Diseases: An Official Publication of the Infectious Diseases Society of America. 2017;65(Suppl 2):S133–S42. 10.1093/cid/cix661 PMC5850429. 29117329 PMC5850429

[2] Goldenberg RL, Culhane JF, Iams JD, Romero R. Preterm birth 1—Epidemiology and causes of preterm birth. Lancet. 2008;371(9606):75–84. 10.1016/S0140-6736(08)60074-4 WOS:000252192600033. 18177778 PMC7134569

[3] Tsiartas P, Holst R, Wennerholm U, Hagberg H, Hougaard D, Skogstrand K, et al Prediction of spontaneous preterm delivery in women with threatened preterm labour: a prospective cohort study of multiple proteins in maternal serum. BJOG: An International Journal of Obstetrics & Gynaecology. 2012;119(7):866–73. 22530716 10.1111/j.1471-0528.2012.03328.x

[4] Goldenberg RL. The management of preterm labor. Obstetrics & Gynecology. 2002;100(5, Part 1):1020–37. 12423870 10.1016/s0029-7844(02)02212-3

[5] Honest H, Bachmann LM, Gupta JK, Kleijnen J, Khan KS. Accuracy of cervicovaginal fetal fibronectin test in predicting risk of spontaneous preterm birth: systematic review. British Medical Journal. 2002;325(7359):301–4C. 10.1136/bmj.325.7359.301 WOS:000177482500015. 12169504 PMC117763

[6] Ramsey PS, Andrews WW. Biochemical predictors of preterm labor: fetal fibronectin and salivary estriol. Clinics in perinatology. 2003;30(4):701–33. 14714920 10.1016/s0095-5108(03)00109-x

[7] Heng YJ, Liong S, Permezel M, Rice GE, Di Quinzio MK, Georgiou HM. Human cervicovaginal fluid biomarkers to predict term and preterm labor. Frontiers in physiology. 2015;6. 10.3389/fphys.2015.00151 PMC4429550 26029118

[8] Liong S, Di Quinzio M, Fleming G, Permezel M, Rice G, Georgiou H. New biomarkers for the prediction of spontaneous preterm labour in symptomatic pregnant women: a comparison with fetal fibronectin. BJOG: An International Journal of Obstetrics & Gynaecology. 2015;122(3):370–9. 25056135 10.1111/1471-0528.12993

[9] Amabebe E, Reynolds S, Stern VL, Parker JL, Stafford GP, Paley MN, et al Identifying metabolite markers for preterm birth in cervicovaginal fluid by magnetic resonance spectroscopy. Metabolomics. 2016;12(4):1–11. 10.1007/s11306-016-0985-x PMC4783437 27065760

[10] Amabebe E, Reynolds S, Stern V, Stafford G, Paley M, Anumba DOC. Cervicovaginal Fluid Acetate: A Metabolite Marker of Preterm Birth in Symptomatic Pregnant Women. Frontiers in Medicine. 2016;3(48). 10.3389/fmed.2016.00048 27777928 PMC5056530

[11] Srinivasan S, Morgan MT, Fiedler TL, Djukovic D, Hoffman NG, Raftery D, et al Metabolic Signatures of Bacterial Vaginosis. mBio. 2015;6(2):e00204–15. 10.1128/mBio.00204-15 25873373 PMC4453549

[12] Lamont RF. Advances in the Prevention of Infection-Related Preterm Birth. Frontiers in immunology. 2015;6(566). 10.3389/fimmu.2015.00566 26635788 PMC4644786

[13] Nelson DB, Hanlon A, Nachamkin I, Haggerty C, Mastrogiannis DS, Liu C, et al Early Pregnancy Changes in Bacterial Vaginosis‐Associated Bacteria and Preterm Delivery. Paediatric and perinatal epidemiology. 2014;28(2):88–96. 10.1111/ppe.12106 24405280 PMC4031320

[14] Foxman B, Wen A, Srinivasan U, Goldberg D, Marrs CF, Owen J, et al Mycoplasma, bacterial vaginosis-associated bacteria BVAB3, race, and risk of preterm birth in a high-risk cohort. American journal of obstetrics and gynecology. 2014;210(3):226.e1–.e2267. Epub 2013/10/04. 10.1016/j.ajog.2013.10.003 . 24096128 PMC3943817

[15] Amabebe E, Chapman DR, Stern VL, Stafford G, Anumba DOC. Mid-gestational changes in cervicovaginal fluid cytokine levels in asymptomatic pregnant women are predictive markers of inflammation-associated spontaneous preterm birth. Journal of Reproductive Immunology. 2018;126:1–10. 10.1016/j.jri.2018.01.001 29367099 PMC5886036

[16] Chan RL. Biochemical markers of spontaneous preterm birth in asymptomatic women. BioMed research international. 2014;2014. 10.1155/2014/164081 PMC3914291 24551837

[17] Georgiou HM, Di Quinzio MK, Permezel M, Brennecke SP. Predicting Preterm Labour: Current Status and Future Prospects. Disease markers. 2015;2015:435014 Epub 2015/07/15. 10.1155/2015/435014 26160993 PMC4486247

[18] Amabebe E. Analysis of cervicovaginal fluid metabolome and microbiome in relation to preterm birth [PhD thesis]. White Rose eTheses Online: University of Sheffield; 2016.

[19] Stafford GP, Parker JL, Amabebe E, Kistler J, Reynolds S, Stern V, et al Spontaneous Preterm Birth Is Associated with Differential Expression of Vaginal Metabolites by Lactobacilli-Dominated Microflora. Frontiers in Physiology. 2017;8(615). 10.3389/fphys.2017.00615 28878691 PMC5572350

[20] Amabebe E, Reynolds S, Stern V, Stafford G, Paley M, Anumba D. Prognostic Capacity of Cervicovaginal Fluid Acetate-Glutamate Ratio for Risk of Preterm Delivery within Two Weeks of Presentation with Symptoms of Preterm Labor. Reproductive Sciences. 2017;24:175A-A. WOS:000399043900390.

[21] Bernhard AE, Field KG. A PCR assay To discriminate human and ruminant feces on the basis of host differences in Bacteroides-Prevotella genes encoding 16S rRNA. Applied and environmental microbiology. 2000;66(10):4571–4. Epub 2000/09/30. 10.1128/aem.66.10.4571-4574.2000 11010920 PMC92346

[22] Ling Z, Kong J, Liu F, Zhu H, Chen X, Wang Y, et al Molecular analysis of the diversity of vaginal microbiota associated with bacterial vaginosis. BMC genomics. 2010;11 10.1186/1471-2164-11-488 WOS:000282790600001. 20819230 PMC2996984

[23] Walter J, Margosch D, Hammes WP, Hertel C. Detection of Fusobacterium species in human feces using genus-specific PCR primers and denaturing gradient gel electrophoresis. Microbial Ecology in Health and Disease. 2002;14(3):129–32. 10.1080/089106002320644294 BCI:BCI200300038741.

[24] Zariffard MR, Saifuddin M, Sha BE, Spear GT. Detection of bacterial vaginosis-related organisms by real-time PCR for Lactobacilli, Gardnerella vaginalis and Mycoplasma hominis. FEMS immunology and medical microbiology. 2002;34(4):277–81. Epub 2002/11/22. 10.1111/j.1574-695X.2002.tb00634.x . 12443827

[25] Jacobsson B, Mattsby-Baltzer I, Hagberg H. Interleukin-6 and interleukin-8 in cervical and amniotic fluid: relationship to microbial invasion of the chorioamniotic membranes. 2005;112(6):719–24. 10.1111/j.1471-0528.2005.00536.x 15924526

[26] Witkin S, Linhares I. Why do lactobacilli dominate the human vaginal microbiota? 2017;124(4):606–11. 10.1111/1471-0528.14390 28224747

[27] Smith SB, Ravel J. The vaginal microbiota, host defence and reproductive physiology. 2017;595(2):451–63. 10.1113/JP271694 27373840 PMC5233653

[28] Vitali B, Cruciani F, Picone G, Parolin C, Donders G, Laghi L. Vaginal microbiome and metabolome highlight specific signatures of bacterial vaginosis. Eur J Clin Microbiol. 2015;34(12):2367–76. 10.1007/s10096-015-2490-y 26385347

[29] Laghi L, Picone G, Cruciani F, Brigidi P, Calanni F, Donders G, et al Rifaximin modulates the vaginal microbiome and metabolome in women affected by bacterial vaginosis. Antimicrobial agents and chemotherapy. 2014;58(6):3411–20. 10.1128/AAC.02469-14 24709255 PMC4068465

[30] Aldunate M, Srbinovski D, Hearps AC, Latham CF, Ramsland PA, Gugasyan R, et al Antimicrobial and immune modulatory effects of lactic acid and short chain fatty acids produced by vaginal microbiota associated with eubiosis and bacterial vaginosis. Frontiers in physiology. 2015;6:164 10.3389/fphys.2015.00164 26082720 PMC4451362

[31] Amabebe E, Anumba DOC. The Vaginal Microenvironment: The Physiologic Role of Lactobacilli. 2018;5(181). 10.3389/fmed.2018.00181 29951482 PMC6008313

[32] Wolfgang Buckel HAB. Two Pathways of Glutamate Fermentation by Anaerobic Bacteria. Journal of Bacteriology. 1974;117 (3):1248–60. 4813895 10.1128/jb.117.3.1248-1260.1974 PMC246608

[33] Ramezani M, Resmer KL, White RL. Glutamate racemization and catabolism in Fusobacterium varium. The FEBS Journal. 2011;278(14):2540–51. 10.1111/j.1742-4658.2011.08179.x 21575137

[34] Gajer P, Brotman RM, Bai GY, Sakamoto J, Schuette UME, Zhong X, et al Temporal Dynamics of the Human Vaginal Microbiota. Sci Transl Med. 2012;4(132). ARTN 132ra52 10.1126/scitranslmed.3003605 ISI:000303596400004. 22553250 PMC3722878

[35] Fanos V, Atzori L., Makarenko K., Melis G.B., Ferrazi E. Metabolomics application in maternal-fetal medicine. BioMed research international. 2013;2013(720514):10.1155/2013/720514. PMC3690726 23841090

[36] Romero R, Mazaki-Tovi S., Vaisbuch E., Kusanovic J.P., Chaiworapongsa T., Gomez R., Nien J.K., Yoon B.H., Mazor M., Luo J., Banks D., Ryals J., Beecher C. Metabolomics in premature labor: a novel approach to identify patients at risk for preterm delivery. J Matern Fetal Neonatal Med. 2010;23(12):1344–59. 10.3109/14767058.2010.482618 20504069 PMC3440243

[37] Hamilton SA, Tower CL, Jones RL. Identification of Chemokines Associated with the Recruitment of Decidual Leukocytes in Human Labour: Potential Novel Targets for Preterm Labour. PLOS ONE. 2013;8(2):e56946 10.1371/journal.pone.0056946 23451115 PMC3579936

[38] Ghartey J, Bastek JA, Brown AG, Anglim L, Elovitz MA. Women with preterm birth have a distinct cervicovaginal metabolome. American Journal of Obstetrics and Gynecology. 2015;212(6):776. e1–.e12. 25827503 10.1016/j.ajog.2015.03.052 PMC4877183

[39] Chow SS, Craig ME, Jones CA, Hall B, Catteau J, Lloyd AR, et al Differences in amniotic fluid and maternal serum cytokine levels in early midtrimester women without evidence of infection. Cytokine. 2008;44(1):78–84. Epub 2008/08/16. 10.1016/j.cyto.2008.06.009 . 18703348

[40] Yang Q, El-Sayed Y, Rosenberg-Hasson Y, Hirschberg DL, Nayak NR, Schilling J, et al Multiple cytokine profile in plasma and amniotic fluid in a mouse model of pre-term labor. Am J Reprod Immunol. 2009;62(5):339–47. Epub 2009/10/09. 10.1111/j.1600-0897.2009.00743.x . 19811468

[41] Athayde N, Romero R, Maymon E, Gomez R, Pacora P, Araneda H, et al A role for the novel cytokine RANTES in pregnancy and parturition. American Journal of Obstetrics and Gynecology. 1999;181(4):989–94. 10.1016/s0002-9378(99)70337-6 10521766

[42] Vrachnis N, Karavolos S, Iliodromiti Z, Sifakis S, Siristatidis C, Mastorakos G, et al Impact of mediators present in amniotic fluid on preterm labour. In Vivo. 2012;26(5):799–812. 22949593

[43] Agrawal V, Hirsch E. Intrauterine infection and preterm labor. Seminars in Fetal and Neonatal Medicine. 2012;17(1):12–9. 10.1016/j.siny.2011.09.001 21944863 PMC3242863

[44] Keelan JA. Pharmacological inhibition of inflammatory pathways for the prevention of preterm birth. Journal of Reproductive Immunology. 2011;88(2):176–84. 10.1016/j.jri.2010.11.003 21236496

[45] Keelan JA. Intrauterine inflammatory activation, functional progesterone withdrawal, and the timing of term and preterm birth. Journal of Reproductive Immunology. 2018;125:89–99. 10.1016/j.jri.2017.12.004 29329080

[46] Kalinka J, Sobala W, Wasiela M, Brzezińska-Błaszczyk E. Decreased Proinflammatory Cytokines in Cervicovaginal Fluid, as Measured in Midgestation, are Associated with Preterm Delivery. American Journal of Reproductive Immunology. 2005;54(2):70–6. 10.1111/j.1600-0897.2005.00289.x 16105098

[47] Inglis SR, Jeremias J, Kuno K, Lescale K, Peeper Q, Chervenak FA, et al Detection of tumor necrosis factor-alpha, interleukin-6, and fetal fibronectin in the lower genital tract during pregnancy: relation to outcome. Am J Obstet Gynecol. 1994;171(1):5–10. Epub 1994/07/01. 10.1016/s0002-9378(94)70069-9 . 8030732

[48] Paternoster DM, Stella A, Gerace P, Manganelli F, Plebani M, Snijders D, et al Biochemical markers for the prediction of spontaneous pre-term birth. 2002;79(2):123–9. 10.1016/S0020-7292(02)00243-6 12427396

[49] Lockwood CJ, Ghidini l, Wein R, Lapinski R, Casal D, Berkowitz RL. Increased interleukin-6 concentrations in cervical secretions are associated with preterm delivery. American Journal of Obstetrics and Gynecology. 1994;171(4):1097–102. 10.1016/0002-9378(94)90043-4 7943078

[50] Kalan AM, Simhan HN. Mid-trimester cervical inflammatory milieu and sonographic cervical length. American Journal of Obstetrics and Gynecology. 2010;203(2):126.e1–.e5. 10.1016/j.ajog.2010.03.013. 20451891

[51] Smith SB, Ravel J. The vaginal microbiota, host defence and reproductive physiology. The Journal of Physiology. 2016:n/a-n/a. 10.1113/JP271694 27373840 PMC5233653

[52] Donders GG, Van Calsteren C, Bellen G, Reybrouck R, Van den Bosch T, Riphagen I, et al Association between abnormal vaginal flora and cervical length as risk factors for preterm birth. Ultrasound in Obstetrics & Gynecology. 2010. 10.1002/uog.7568 20104531

[53] Agrawal V, Hirsch E, editors. Intrauterine infection and preterm labor Seminars in Fetal and Neonatal Medicine; 2012: Elsevier. 10.1016/j.siny.2011.09.001 PMC3242863 21944863

[54] Holst RM, Hagberg H, Wennerholm UB, Skogstrand K, Thorsen P, Jacobsson B. Prediction of spontaneous preterm delivery in women with preterm labor: analysis of multiple proteins in amniotic and cervical fluids. Obstet Gynecol. 2009;114(2 Pt 1):268–77. Epub 2009/07/23. 10.1097/AOG.0b013e3181ae6a08 . 19622987

[55] Vogel I, Goepfert AR, Thorsen P, Skogstrand K, Hougaard DM, Curry AH, et al Early second-trimester inflammatory markers and short cervical length and the risk of recurrent preterm birth. J Reprod Immunol. 2007;75(2):133–40. Epub 2007/04/20. 10.1016/j.jri.2007.02.008 . 17442403

[56] Deshpande S, van Asselt A, Tomini F, Armstrong N, Allen A, Noake C, et al Rapid fetal fibronectin testing to predict preterm birth in women with symptoms of premature labour: a systematic review and cost analysis. 2013. 10.3310/hta17400 PMC4781038 24060096

[57] Boots AB, Sanchez-Ramos L, Bowers DM, Kaunitz AM, Zamora J, Schlattmann P. The short-term prediction of preterm birth: a systematic review and diagnostic metaanalysis. American Journal of Obstetrics and Gynecology. 2014;210(1):54.e1–.e10. 10.1016/j.ajog.2013.09.004. 24021995

