# Supplementary figures and images for: Infection/inflammation-associated preterm delivery within 14 days of presentation with symptoms of preterm labour: A multivariate predictive model
Source: PLoS One. 2019 Sep 12;14(9):e0222455. doi: 10.1371/journal.pone.0222455 (PMC6742395; doi:10.1371/journal.pone.0222455)

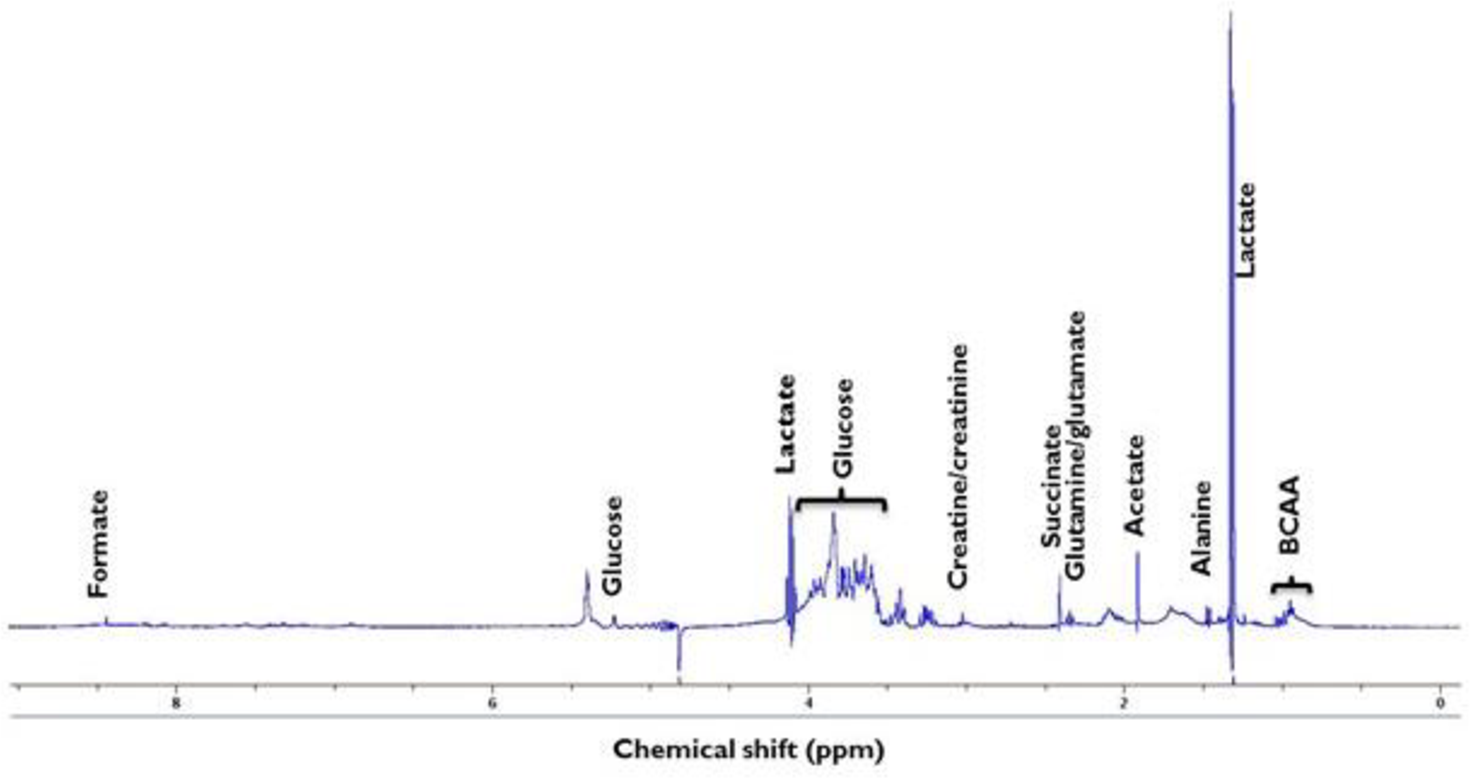

Supplement: S1 Fig — BCAA, branched chain amino acids; ppm, parts per million. (TIF) [file pone.0222455.s001.tif]
