# Supplementary material for: Infection/inflammation-associated preterm delivery within 14 days of presentation with symptoms of preterm labour: A multivariate predictive model
Source: PLoS One. 2019 Sep 12;14(9):e0222455. doi: 10.1371/journal.pone.0222455 (PMC6742395; doi:10.1371/journal.pone.0222455)
Supplement: S1 Table — GBS, group B Streptococcus. (PDF) [file pone.0222455.s002.pdf]

Primer	Sequence (5'-3')	Target	Annealing temp (ºC)	Amplicon size (bp)	Source
LABF<br>LABR	AGAGTTTGATYMTGGCTCAG<br>CACCGCTACACATGGAG	*Lactobacillus*	62	667	[22]
LJ2F<br>LJ2R	GCACGTAGTTAGCCGTGACT<br>GAAACAGATGCTAATACCGG	*L. jensenii*	61	~350	This study
LBF<br>LBR	ATGGAAGAACACCAGTGGCG<br>CAGCACTGAGAGGCGGAAAC	*L. jensenii*<br>*L. crispatus*	50	~150	[24]
FBF<br>FBR	ACTCCTACGGGAGGCAGCAGT<br>CGAATTTCACCTCTACACTTGT	*Fusobacterium*	60	341	[23]
GV1F<br>GV3R	GGAAACGGGTGGTAATGCTGG<br>CGAAGCCTAGGTGGGCCATT	*G. vaginalis*	65	125	[58]
BAC32F<br>BAC708R	AACGCTAGCTACAGGCTT<br>CAATCGGAGTTCTTCGTG	*Bacteroides-Prevotella*	53	676	[21]
Sag59<br>Sag190	TTTCACCAGCTGTATTAGAAGTA<br>GTTCCCTGAACATTATCTTTGAT	*GBS*	55	153	[59]
M. curt-440F<br>M. curt-1026R	TTCTCGCGAAAAAGGCACAG<br>CTGGCCCATCTCTGGAACCA	*M. curtisii*	57	586	[60]
Mobil-577F<br>M.mulie-1026R	GCTCGTAGGTGGTTCGTCGC<br>CCACACCATCTCTGGCATG	*M. mulieris*	62	449	[60]
Mh1-F<br>Mh2-R	CAATGGCTAATGCCGGATACGC<br>GGTACCGTCAGTCTGCAAT	*M. hominis*	62	334	[24]
