# Supplementary material for: Infection/inflammation-associated preterm delivery within 14 days of presentation with symptoms of preterm labour: A multivariate predictive model
Source: PLoS One. 2019 Sep 12;14(9):e0222455. doi: 10.1371/journal.pone.0222455 (PMC6742395; doi:10.1371/journal.pone.0222455)
Supplement: S2 Table — AUC, area under the ROC curve; CI, confidence interval; Sens, sensitivity; Spec, specificity; PPV, positive predictive value; NPV, negative predictive value; +LR, positive likelihood ratio; -LR, negative likelihood ratio; BMI, body mass index; CL, cervical length; qfFN, quantitative fetal fibronectin; Ace/Glxconc, ratio of acetate to glutamate concentrations. All metabolite concentrations were measured by enzyme-based spectrophotometry. (PDF) [file pone.0222455.s003.pdf]

Biomarkers	AUC (95%CI)	Sens (%)	Spec (%)	PPV (%)	NPV (%)	LR+	LR-	*P*-value
**< 37 weeks' gestation**								
RANTES	0.87 (0.60-0.99)	83.3	88.9	83.3	88.9	2.5	0.5	< 0.001
Acetate	0.79 (0.66-0.89)	66.7	82.9	58.8	87.2	3.9	0.4	< 0.001
CL	0.79 (0.66-0.89)	83.3	73.8	45.5	93.9	3.2	0.2	0.001
qfFN	0.78 (0.65-0.88)	50.0	100.0	100.0	90.0		0.5	0.002
Ace/Glx_{conc}	0.74 (0.60-0.85)	60.0	81.0	52.9	85.0	3.2	0.5	0.001
IL-6	0.72 (0.59-0.83)	92.9	42.6	32.5	95.2	1.6	0.2	0.007
TNF-r1	0.70 (0.59-0.79)	63.2	74.7	38.7	88.9	2.5	0.5	0.007
BMI	0.70 (0.58-0.80)	92.9	43.3	27.7	96.3	1.6	0.2	0.01
L/D-lactate ratio + Acetate + IL-6 + TNF-r1	0.82 (0.65-0.93)	77.8	79.2	58.3	90.5	3.7	0.3	0.001
Acetate + IL-6 + TNF-r1	0.84 (0.67-0.94)	80.0	84.0	66.7	91.3	5.0	0.2	< 0.001
Total lactate + Acetate + IL-6 + TNF-r1	0.85 (0.69-0.95)	90.0	72.0	56.2	94.7	3.2	0.1	< 0.001
**Delivery within 2 weeks**								
RANTES	0.91 (0.65-1.0)	100.0	81.8	66.7	100.0	5.5	0.0	< 0.001
IL-6	0.79 (0.67-0.88)	62.5	90.6	50.0	94.1	6.6	0.4	0.001
Acetate	0.73 (0.59-0.84)	62.5	89.6	50.0	93.5	6.0	0.4	0.04
Ace/Glx_{conc}	0.74 (0.61-0.85)	87.5	63.3	28.0	96.9	2.4	0.2	0.006
Maternal age	0.73 (0.62-0.82)	77.8	64.1	20.0	96.2	2.2	0.4	0.02
Maternal age + Ace/Glx + L/D-lactate ratio	0.84 (0.67-0.94)	66.7	92.9	66.7	92.9	9.3	0.4	< 0.001
L/D-lactate ratio + Ace/Glx_{conc} + IL-6	0.84 (0.67-0.94)	83.3	82.1	50.0	95.8	4.7	0.2	0.001
Total lactate + Ace/Glx_{conc} + IL-6	0.82 (0.66-0.93)	66.7	93.3	66.7	93.3	10.0	0.4	0.002

Ace/Glx$_{conc}$ + IL-6	0.82 (0.65-0.93)	83.3	73.3	38.5	95.7	3.1	0.2	0.003
