# Supplementary material for: Infection/inflammation-associated preterm delivery within 14 days of presentation with symptoms of preterm labour: A multivariate predictive model
Source: PLoS One. 2019 Sep 12;14(9):e0222455. doi: 10.1371/journal.pone.0222455 (PMC6742395; doi:10.1371/journal.pone.0222455)
Supplement: S1 File — (PDF) [file pone.0222455.s004.pdf]

S1 File. CVF cytokine measurement

From the 400 µl CVF sample reserved for bacterial DNA extraction, 50 µl was aspirated and transferred into a new 1.5 µl microfuge tube and analysed for IL-1α, IL-1β, IL-2, IL-4, IL-6, IL-8, IL-10, IL-12p20, RANTES, TNF-r1, and IFN-γ by multiplexed bead-based immunoassay (BDTM Cytometric Bead Array, BD Biosciences, CA, USA). This was performed according to the BD CBA Human Soluble Protein Master Buffer Kit instruction. Twenty-five microliters of each sample were pipetted into ELKAY 1.1 ml and the Standard component in the kit was lyophilised and transferred to a 15 ml polypropylene tube for reconstitution. After reconstitution in 4.0 ml Assay Diluent, the standards for each cytokine had a concentration of 2500 pg/ml, except TNF-r1 = 10000 pg/ml (Top Standard). The standard solution was left to equilibrate for at least 30 min before making any dilution. 25 µl of assay diluent was subsequently added to each tube labelled 1:2, 1:4, 1:8, 1:16: 1:32, 1:64, 1:128, 1:256. Doubling dilutions were then performed by transferring 25 µl from the Top standard to the 1:2 dilution tube and thoroughly mixed before continuation to the next tube. The process was continued until 1:256 tube was reached. The first tube, which contained assay diluent only, was used as Bottom Standard and served as the 0 pg/ml control tube. A solution of capture beads-antibodies conjugate was prepared and 25 µl of the solution was added to each tube containing standards and samples. The tube content was then gently mixed and incubated at room temperature shielded from ultraviolet light for one hour. This was to allow for adequate binding of the capture antibodies to the cytokines in the samples. After an hour of suspension and binding, 25 µl of mixed Phycoerythrin detection reagent was added to the tubes and incubated for additional 2 hours to allow the formation of a Sandwich complexes (organometallic compounds) at room temperature. The samples were then washed, centrifuged individually, left for resuspension, and then transferred to the plate. Mean Fluorescence Intensity (MFI) were generated by a Life Technologies Attune Acoustic Focusing Cytometry and Attune NxT Cytometric Software v.2.1. Actual concentration of each cytokine in each sample was extrapolated from the generated Standard curve. Cytokine analyses were performed by staff blinded from patient's clinical data and eventual delivery outcome.
